# The Empty Bladder is Preferred in Some Cases of Radiation Therapy for Rectal Cancer

**DOI:** 10.1016/j.adro.2025.101872

**Published:** 2025-10-03

**Authors:** Inna Ospovat, Albert Schlocker, Natan Shtraus, Ravit Geva, Shani Hazan, Tatyana Shevchuk, Alexander Barenboim, Ido Wolf, Orit Gutfeld, Viacheslav Soyfer

**Affiliations:** Tel Aviv Sourasky Medical Center, Tel Aviv University, Tel Aviv, Israel

## Introduction

Radiation therapy is an integral component in the treatment of rectal cancer. Appropriate positioning of patients for the best dosimetric coverage of the treatment volumes that minimizes the irradiation of normal structures, especially avoiding the small bowel, is a primary task for physicians in the simulation and treatment planning of rectal cancer radiation therapy. Many studies on the preference for either prone or supine positioning exist in the literature. Generally, the supine position is preferable in terms of treatment reproducibility, while the prone position is better for small bowel radiation avoidance.^1^, ^2^, ^3^ A full bladder is usually prescribed in order to displace the attached small bowel away from the radiation treatment fields.^4^, ^5^, ^6^

The rectouterine space in women, called the Pouch of Douglas, is the lowest part of the abdominal cavity. In men, this low space is the rectovesical space.^7^ Normally, this cavity contains a small amount of peritoneal fluid. The small bowel can transiently enter this lower part of the abdominal cavity in certain situations, notably when the patient is in an upright or supine position. An increase in abdominal pressure may also cause the small bowel to translocate to the rectouterine or rectovesical space. Moreover, pelvic inflammatory disease, previous surgery, or an enlarged myomatous uterus can lead to a constant intestinal presence in these cavities. The current standard of care for radiation therapy for rectal cancer, if indicated, is administered prior to surgery, either by 3-dimensional conformal or volumetric modulated arc therapy technique.^8^ The treatment fields typically include the rectum and surrounding lymphatics, and according to contouring atlases, the posterior wall of the bladder or uterus is included.^9^

## Case Description

In this clinical report, a 63-year-old otherwise healthy woman diagnosed with adenocarcinoma of the mid-rectum T3N1 was simulated for preoperative radiation therapy. Traditional measures to eliminate entrapped small bowel loops between the rectum and bowel were not effective. The standard simulation included an oral contrast in both supine and prone positioning, including the use of a belly board and a full bladder. It appeared that in both supine and prone orientations, there was a significant volume of small bowel loops entrapped behind the bladder.

**Figure 1:**
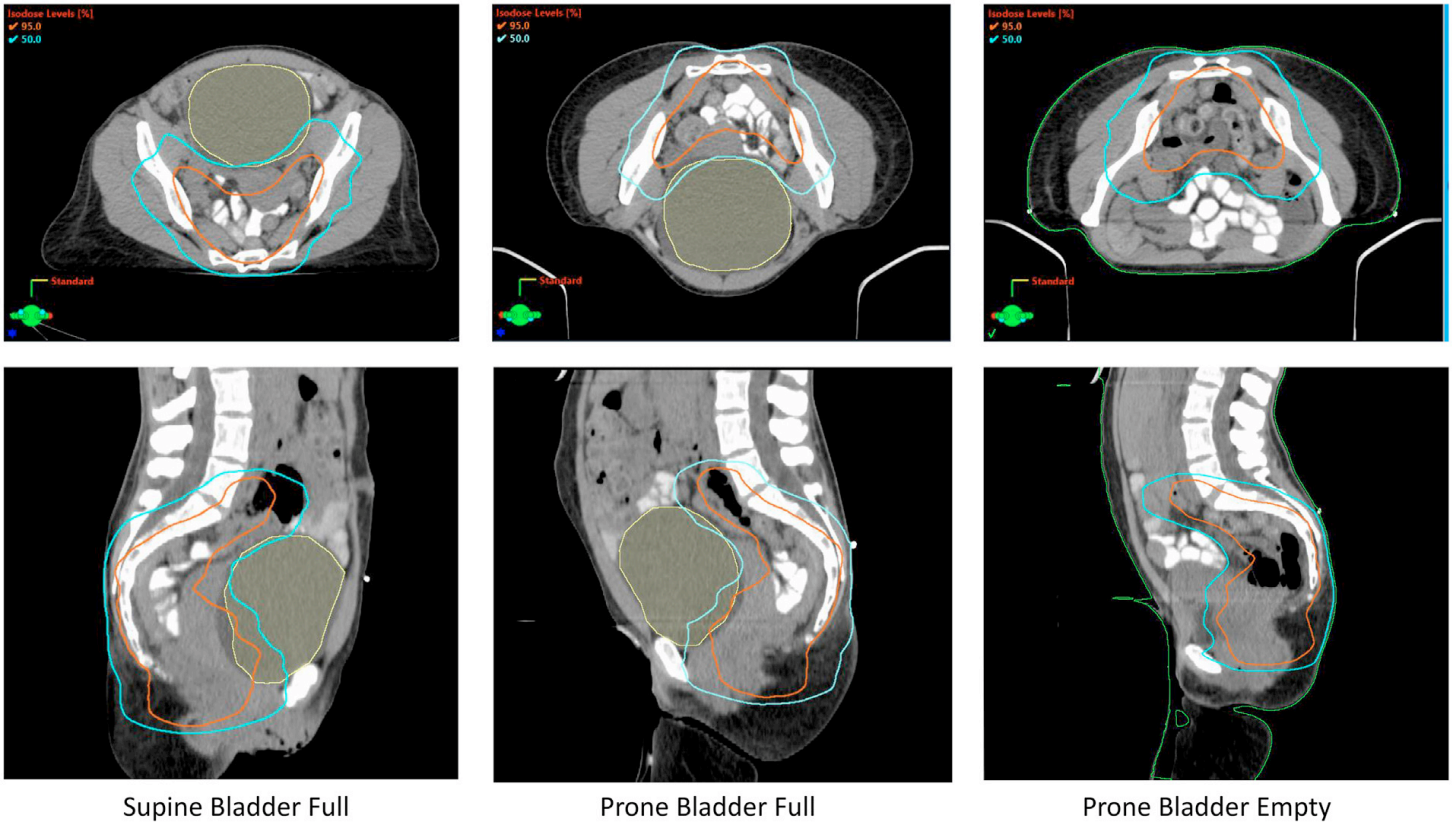
Volumetric modulated arc therapy treatment plans (transverse and sagittal views) discussed in the article for both supine and prone orientations, with a full and empty bladder, illustrate that for this patient, the lowest dose to the small bowel was achieved with the patient prone and an empty bladder.

A third simulation scan, with the patient prone and an empty bladder, allowed the release of small bowel anteriorly and enabled treatment planning with minimal small bowel exposure (Fig. 1).

## Conclusion

In summary, computed tomography simulation of rectal patients in the prone position concurrent with an empty bladder could be considered in cases when the small bowel is located in the low pelvis behind the bladder or uterus, or behind the bladder in men.

## Disclosures

The authors declare that they have no known competing financial interests or personal relationships that could have appeared to influence the work reported in this paper.
